# Integrated Chemical and Transcriptomic Analysis Reveals the Distribution of Protopanaxadiol- and Protopanaxatriol-Type Saponins in *Panax notoginseng*

**DOI:** 10.3390/molecules23071773

**Published:** 2018-07-19

**Authors:** Guangfei Wei, Fugang Wei, Can Yuan, Zhongjian Chen, Yong Wang, Jiang Xu, Yongqing Zhang, Linlin Dong, Shilin Chen

**Affiliations:** 1 Shandong University of Traditional Chinese Medicine, Jinan 250355, China; 15098855936@163.com (G.W.); zyq622003@126.com (Y.Z.); 2 Key Laboratory of Beijing for Identification and Safety Evaluation of Chinese Medicine, Institute of Chinese Materia Medica, China Academy of Chinese Medical Sciences, Beijing 100700, China; scnkyjzsxy@163.com (C.Y.); jxu@icmm.ac.cn (J.X.); 3 Wenshan Miaoxiang Notoginseng Technology Co., Ltd., Wenshan 663000, China; weifugang@live.com; 4 Institute of Sanqi Research, Wenshan University, Wenshan 663000, China; panaxnotoginseng@126.com (Z.C.); ws-wangyong37@163.com (Y.W.)

**Keywords:** *Panax notoginseng*, different parts, transcriptome analysis, protopanaxadiol-type saponins, protopanaxatriol-type saponins, CYP genes

## Abstract

*Panax notoginseng* is famous for its important therapeutic effects and commonly used worldwide. The active ingredients saponins have distinct contents in different tissues of *P. notoginseng*, and they may be related to the expression of key genes in the synthesis pathway. In our study, high-performance liquid chromatography results indicated that the contents of protopanaxadiol-(Rb1, Rc, Rb2, and Rd) and protopanaxatriol-type (R1, Rg1, and Re) saponins in below ground tissues were higher than those in above ground tissues. Clustering dendrogram and PCA analysis suggested that the below and above ground tissues were clustered into two separate groups. A total of 482 and 882 unigenes were shared in the below and above ground tissues, respectively. A total of 75 distinct expressions of CYPs transcripts (RPKM ≥ 10) were detected. Of these transcripts, 38 and 37 were highly expressed in the below ground and above ground tissues, respectively. RT-qPCR analysis showed that CYP716A47 gene was abundantly expressed in the above ground tissues, especially in the flower, whose expression was 31.5-fold higher than that in the root. CYP716A53v2 gene was predominantly expressed in the below ground tissues, especially in the rhizome, whose expression was 20.1-fold higher than that in the flower. Pearson’s analysis revealed that the CYP716A47 expression was significantly correlated with the contents of ginsenoside Rc and Rb2. The CYP716A53v2 expression was associated with the saponin contents of protopanaxadiol-type (Rb1 and Rd) and protopanaxatriol-type (R1, Rg1, and Re). Results indicated that the expression patterns of CYP716A47 and CYP716A53v2 were correlated with the distribution of protopanaxadiol-type and protopanaxatriol-type saponins in *P. notoginseng*. This study identified the pivotal genes regulating saponin distribution and provided valuable information for further research on the mechanisms of saponin synthesis, transportation, and accumulation.

## 1. Introduction

*Panax notoginseng* (Burk) F. H. Chen is a widely used herbal medicine with important pharmacological effects, such as anti-hypertensive, neuroprotective, anti-inflammatory estrogen-like, anti-atherosclerotic, anti-tumor, and hepatoprotective activities [1,2,3,4]. The medicinal properties of *P. notoginseng* are attributed to its bioactive constituents, namely, triterpene saponins, which are also known as ginsenosides. These saponins can be categorized into two groups based on the skeleton of their aglycones, namely, dammarane-type and oleanane-type saponins [5,6]. Dammarane-type saponins commonly include notoginsenoside R1 and ginsenosides Rb1, Rb2, Rc, Rd, Re, Rd, Rf, and Rg1 [7]. Oleanane-type saponins mainly comprise ginsenosides Ro and Roa [8]. Different parts of *P. notoginseng* contain various types of saponins and show diverse pharmacological activities [3,9]. For example, *P. notoginseng* roots have been widely used to treat cardiovascular diseases and other disorders [10]. *P. notoginseng* leaves are used to cure insomnia and alleviate anxiety [4]. *P. notoginseng* flowers have beneficial effects on hypertension, tinnitus, and vertigo therapy [11,12]. These results indicate that the parts of *P. notoginseng* elicit different therapeutic effects. The identification of the types and contents of these different parts is essential for its safe application in medicinal and health care products.

The types and contents of saponin are correlated with the expression of key genes in the synthesis pathway [13]. Studies have reported that triterpene saponins are synthesized through the mevalonate (MVA) and 2-C-methyl-D-erythritol-4-phosphate (MEP) pathways, and multiple genes are involved in saponin synthesis [14,15]. Isoprenyl diphosphate and dimethylally pyrophosphate produced in these two pathways are then transformed to geranyl pyrophosphate (GPP) by geranyl diphosphatesynthase (GDPS), the formed GPP is then transformed into farnesyl pyrophosphate by farnesyl diphosphate synthase (FPS) [16]. Two FPP (C_15_) molecules yield a C_30_ isoprenoid squalene by squalene synthase (SS) [17]. The formed squalene is catalyzed to produce 2,3-oxidosqualene by squalene epoxidase (SE) [18]. 2,3-Oxidosqualene is catalyzed by dammarenediol-II synthase (DS) to form dammarenediol-II and subsequently undergo cytochrome (CYP) hydroxylation and UDP-glycosyltransferases (UGTs) [19]. FPS, SS, SE, and DS genes show tissue-specific expression patterns, which are correlated with saponin contents [13,15]. CYP716 subfamily genes, namely, CYP716A47 and CYP716A53v2, participate in dammarane-type triterpenoid production, and CYP716A52v2 is implicated in oleanane-type triterpenoid production in *P. ginseng* [20]. CYP716A47 hydroxylates dammarenediol-II at the C-12 position to yield protopanaxadiol in *P. ginseng* [21]. CYP716A53v2 catalyzes protopanaxadiol at the C-6 position to generate protopanaxatriol in *P. ginseng* [22]. CYP716A52v2 acts as β-amyrin 28-oxidase that modifies β-amyrin into oleanolic acid in *P. ginseng* [23]. These results indicate that CYPs play important roles in dammarane-type triterpenoid synthesis in *P. ginseng*. However, reports regarding the expression patterns of CYP genes related to the distribution of protopanaxadiol- and protopanaxatriol-type saponins in different parts of *P. notoginseng* are limited. 

This study aimed to identify the main genes regulating the distribution of protopanaxadiol- and protopanaxatriol-type saponins in different parts of *P. notoginseng* based on the integrated analysis of high-performance liquid chromatography (HPLC) and transcriptome. First, the types and contents of saponins in different tissues of *P. notoginseng* were detected through HPLC. Subsequently, the transcriptome of the six parts was examined using Illumina HiSeq 4000 to identify the key genes related to saponin distribution. RT-qPCR analysis was performed to further confirm the main gene expression patterns in the tissues of *P. notoginseng*. Pearson’s analysis was carried out to state the relationship between saponin contents and gene expression relative to the regulating saponin distribution. 

## 2. Results

### 2.1. Analysis of Saponin Contents in P. notoginseng

**Figure 1:**
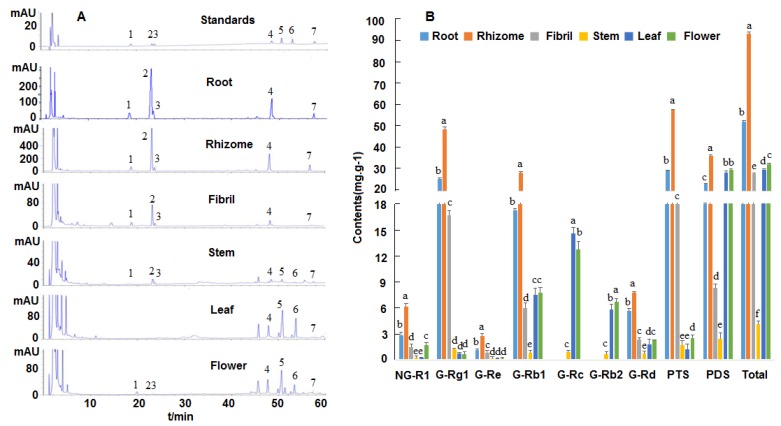
HPLC chromatograms and contents of saponins in *P. notoginseng*. (**A**) HPLC chromatogram profiles. 1. Notoginseng R1; 2. Ginsenoside Rg1; 3. Ginsenoside Re; 4. Ginsenoside Rb1; 5. Ginsenoside Rc; 6. Ginsenoside Rb2; 7. Ginsenoside Rd. (**B**) Contents of the seven saponins. NG: notoginsenoside; G: ginsenoside; PTS: Total amount of protopanaxatriol saponins (NG-R1, G-Rg1, and G-Re); PDS: Total amount of protopanaxadiol saponins (G-Rb1, Rc, Rb2, and Rd); Total: Total amount of protopanaxatriol and protopanaxadiol saponins. Different letters represent the significant differences between the five tissues at *p* < 0.05.

The analysis method was provided in Supplementary Table S1. The contents of the seven saponins were higher in the below ground tissues than in the above ground tissues (Figure 1). R1, Rg1, Re, Rb1, and Rd showed the highest levels in the rhizome (6.16, 48.42, 2.80, 28.13, and 7.73 mg·g^−1^, respectively), followed by those in the root. The contents of Rc and Rb2 were high in the leaf (27.99 mg·g^−1^) and flower (29.45 mg·g^−1^) but were undetected in the below ground tissues (rhizome, root, and fibril). The contents of the total protopanaxatriol saponins (PTS) (NG-R1, G-Rg1, and G-Re) showed high levels in the below ground tissues, especially in the rhizome (57.38 mg·g^−1^) and root (29.09 mg·g^−1^). The contents of the total protopanaxadiol saponins (PDS) (G-Rb1, Rc, Rb2, and Rd) showed higher levels in the below ground tissues than in the above ground tissues. These results indicated that the contents of five saponins (R1, Rg1, Re, Rb1 Rd) and the total saponins were high in the below ground tissues, but Rc and Rb2 were only detected in the above ground tissues.

### 2.2. Transcriptome Analysis in P. notoginseng

Illumina Hiseq sequencing technology was employed to analyze the transcriptome of the 18 samples (six tissues, namely, flower, leaf, stem, rhizome, root, and fibril; three biological replicates for each part) and to clarify the mechanism of saponin synthesis in *P. notoginseng*. A total of 120.70 Gb of data were obtained, and the average output of each sample was 6.71 Gb of data. After filtering was performed, 805 M clean reads with an average size of 150 bp were used for the *de novo* assembly. Overall, 1,325,735 contigs with an average size of 846 bp were obtained, 158,551 unigenes were assembled, and the percentage of GC was 41.05% with an average contig size of 1274 bp and an N50 contig size of 2012 bp (Table 1 and Supplementary Table S2).

### 2.3. Functional Annotation of the Transcriptome

Distinct gene sequences were searched using BLAST with a cutoff E-value of 10^−5^ against public databases, including Nr, KEGG, COG, and SWISS-PROT. A total of 47,184 unigenes (40.6%) returned a significant Blast result through this approach. Among the 116,214 unigenes, approximately 11,079 (9.5%) showed significant matches with the Nr database. A total of 1082; 1816; and 684 unigenes had significant matches with the KEGG, COG, and SWISS-PROT databases, respectively (Supplementary Figure S1).

GO assignments were used to classify the functions of the predicted *P. notoginseng* genes (Supplementary Figure S2A). A total of 367,640 sequences could be categorized into three major GO categories based on sequence homology, namely, biological process, cellular component, and molecular function. They were then classified into 55 functional groups. Cellular, metabolic, and single-organism processes were the most abundant GO terms in the biological process category. Cell and cell part were the most highly dominant terms in the cellular component category. Binding and catalytic activity were the most highly represented terms in the molecular function category. In addition, 35,921 sequences were annotated as a metabolic process category, suggesting that these genes were involved in the metabolite synthesis pathways.

All the unigenes obtained in the present study were subjected to a search against the COG database to predict and classify the possible functions of transcripts (Supplementary Figure S2B). A total of 90,920 unigenes were clustered into 25 function categories. Among the different COG classes, the major COG category was “general functional prediction only” (15,129), followed by “transcription” (8430), “replication, recombination, and repair” (7715), “translation, ribosomal structure, and biogenesis” (6831), “posttranslational modification, protein turnover, and chaperones” (6716), and “signal transduction mechanisms” (6305). The least-represented groups were “nuclear structure” (100) and “extracellular structures” (22). Interestingly, our dataset showed that 2233 uni-transcripts annotated with the COG database represented the “secondary metabolite biosynthesis, transport, and catabolism” category, indicating that a large number of secondary metabolites existed in *P. notoginseng*.

**Figure 2:**
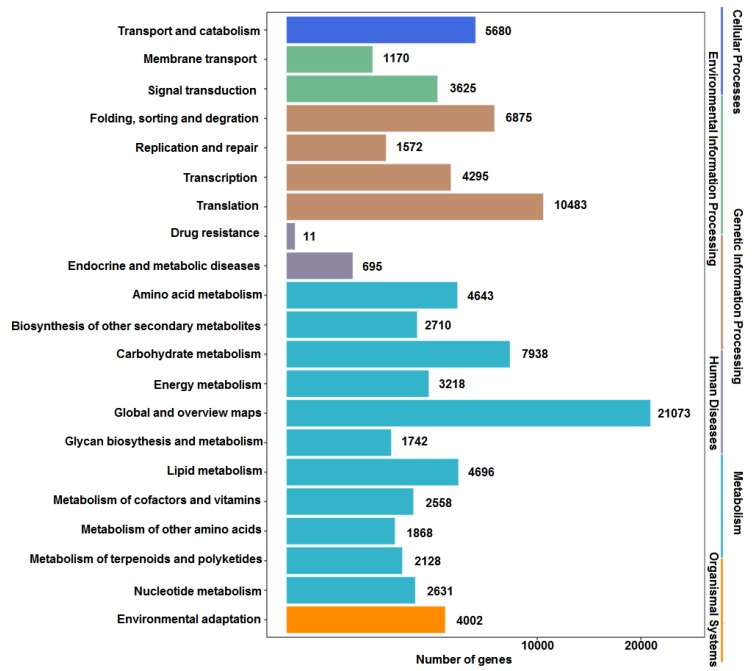
Functional classification of unigenes based on the KEGG pathway.

The annotated sequences were also mapped to the KEGG to identify the biological pathways of *P. notoginseng* (Figure 2). As a result, 93,620 sequences were assigned to 21 KEGG pathways. The pathways with the most represented sequences were metabolism, followed by genetic information processing, cellular process, environmental information processing, organismal system, and human diseases. A total of 55,205 sequences were assigned to the metabolic pathways, including global and overview maps (21,073), carbohydrate metabolism (7938), lipid metabolism (4696), and amino acid metabolism (4643). Furthermore, a total of 2128 sequences were annotated to the metabolism of terpenoids and polyketides. These annotations provided useful information to investigate the specific functions and pathways in *P. notoginseng*.

### 2.4. Differential Expression of Transcripts in the Six tissues of P. notoginseng

**Figure 3:**
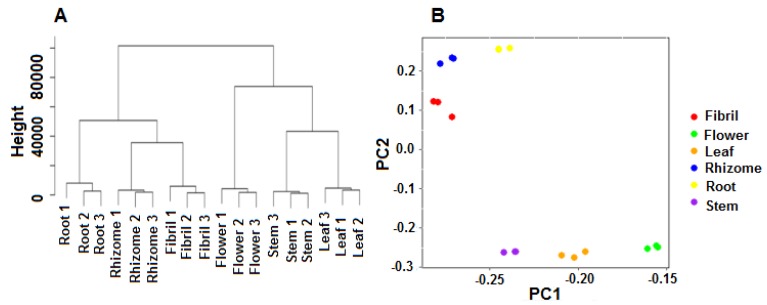
Clustering and PCA analyses of gene expression in six tissues of *P. notoginseng*. A. Clustering tree of gene expression in six tissues of *P. notoginseng*. B. PCA of gene expression in six tissues of *P. notoginseng*.

Cluster dendrogram and PCA analysis were constructed in accordance with the FPKM value, which revealed the different transcripts between below and above ground tissues in *P. notoginseng* (Figure 3).

The below ground and above ground tissues of *P. notoginseng* were clustered into two separate groups, and each part was assembled into the same branches based on the results of the clustering tree (Figure 3A). The PCA results showed that the below ground and above ground tissues were clearly separated in the PC2 axis (Figure 3B).

**Figure 4:**
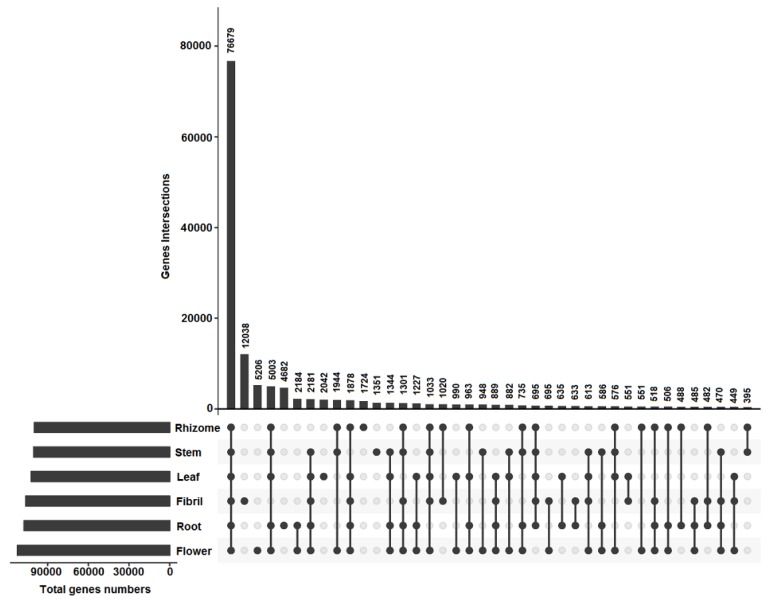
Gene intersection matrix profiles in six tissues of *P. notoginseng*.

The tissue-specific expression genes were calculated to reveal the difference in the transcripts of various tissues of *P. notoginseng* (Figure 4). A total of 143,185 unigenes were applied to create gene intersection profiles. A total of 76,679 unigenes were shared among the six tissues. A total of 482 and 882 unigenes were shared in the below ground and above ground tissues, respectively. A total of 1351; 1724; 2042; 4668; 5206; and 12,038 unigenes were specifically expressed in the stem, rhizome, leaf, root, flower, and fibril, respectively. A large number of DEGs were found in the six tissues of *P. notoginseng* according to the results of the DEGs between two tissues with FC ≥ 2 and FDR ≥ 0.05 (Supplementary Table S3). In comparison with those in rhizome, 1238; 13,983; 19,374; 16,372; and 21,897 DEGs were found in the samples of root, fibril, stem, leaf, and flower, respectively. This result suggested that the transcripts of the six tissues had different expression levels in *P. notoginseng*.

### 2.5. Analysis of Genes Involved in Triterpenoid Saponin Biosynthesis

**Figure 5:**
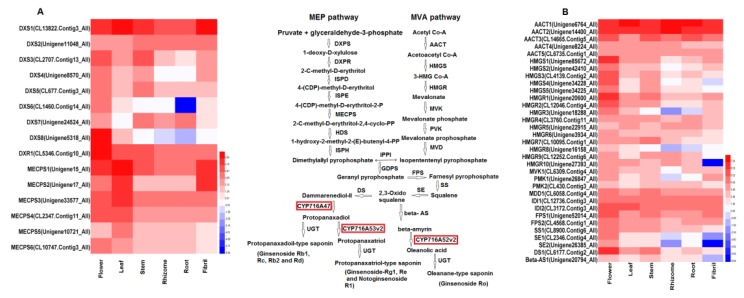
Heatmap of MEP and MVA pathway genes involved in saponin biosynthesis in *P. notoginseng* using an average value of three duplicates (FPKM ≥ 10). A. Transcript abundance profiles of genes in the MEP pathway. B. Transcript abundance profiles of genes in the MVA pathway.

**Figure 6:**
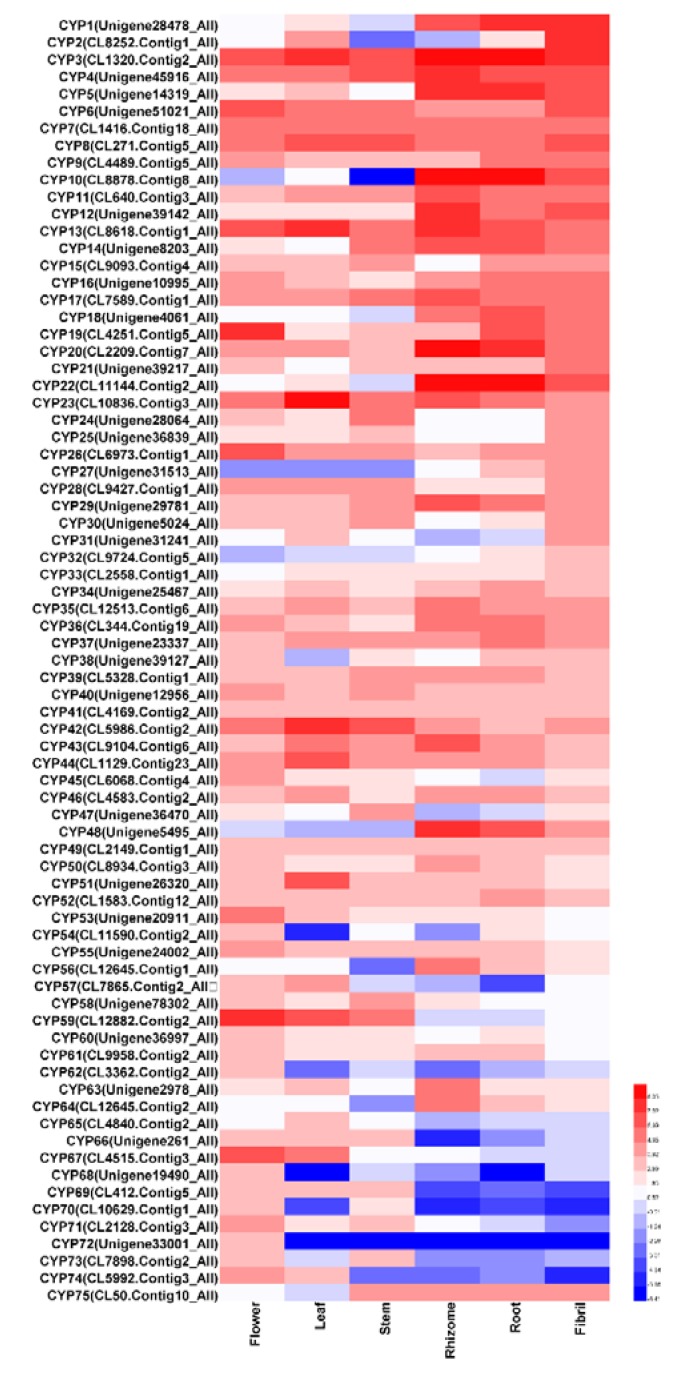
Heatmap of CYP gene expression involved in saponin biosynthesis in *P. notoginseng.*

A total of 16 genes (175 transcripts) were identified to encode the known enzymes involved in saponin biosynthesis through the MVA and MEP pathways based on the analysis of transcriptome (FPKM ≥ 10) (Figure 5 and Figure 6 and Supplementary Tables S4 and S5). The following major genes that were related to saponin synthesis were also identified: FPSs (2), SS (1), SEs (2), DDS (1), and Beta-AS (1).

Transcripts that participated in the MVA pathway had remarkable differential expression in the six tissues at FPKM ≥ 10 (Figure 5B). The expression of the transcripts that encoded HMGR, HMGS, PMK, MVK, FPS, DS, SS, and SE was higher in the above ground tissues than in the below ground tissues, and the highest expression was detected in the flower. Overall, one HMGS (Unigene85672_All), one HMGR (Unigene20600_All), one MVK (CL6309. Contig4_All), two FPSs (Unigene52014_All, CL4568. Contig1_All), and two SEs (CL2346. Contig4_All, CL6177. Contig2_All) were significantly expressed to a greater extent in the flower than in the other tissues. Two transcripts encoding AACT (Unigene6764_All, Unigene14400_All) were highly expressed in the rhizome, and one transcript (CL14665.Contig5_All) was highly expressed in the root. The expression of the MEP and other saponin biosynthesis pathways was similar to that of the MVA pathway (Figure 5A). Among the eight highly abundant transcripts encoding DXS, four transcripts (CL2707. Contig 13_All, Unigene 8570_All, CL1460. Contig 14_All, and Unigene 5318_All) were highly expressed in the flower, and one transcript (CL13822. Contig3_All) was highly expressed in the leaf. One transcript (CL5346. Contig10_All) encoding DXR was highly expressed in the flower. Five transcripts (Unigene15_All, Unigene17_All, Unigene33577_All, CL2347. Contig11_All, and Unigene10721_All) encoding MECPS were highly expressed in the leaf, and one transcript (CL10747. Contig3_All) was highly expressed in the flower.

### 2.6. Analysis of Putative Genes Involved in Saponin Distribution in P. notoginseng

**Figure 7:**
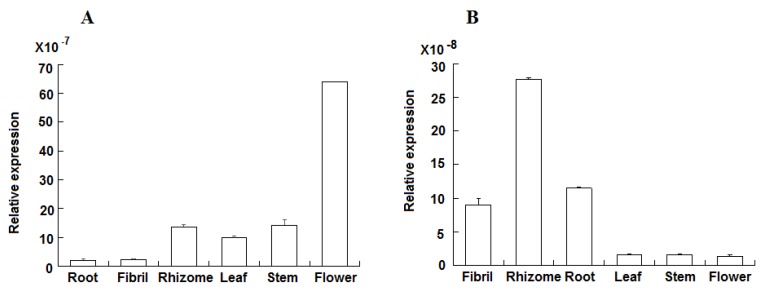
Relative expression of CYP716A47 and CYP716A53v2 genes detected through RT-qPCR. (**A**) CYP716A47; (**B**) CYP716A53v2.

Among the synthetic pathways, specific gene families, such as CYP and UGT, enriched the diversity of saponins. A total of 75 expressed CYP transcripts had distinct expression in our samples (FPKM ≥ 10) (Figure 6). A total of 38 CYPs were highly expressed in the below ground tissues, and the expression of 19 CYPs were the highest in the rhizome. A total of 37 CYPs were highly expressed in the above ground tissues, and the expression of 21 CYPs were the highest in the flower. In addition, one PDS (CL6973. Contig1_All) and two PTS (Unigene8203_All, CL50. Contig10_All) transcripts were identified in our CYP transcript data and annotated as CYP716A47 and CYP716A53v2, respectively. The PDS transcript was expressed in six tissues of *P. notoginseng*, and their expression was the highest in the flower. However, two PTS transcripts were more highly expressed in the rhizome and the root than those in the above ground tissues. Real time-qPCR was performed to detect the expression values of CYP716A47 and CYP716A53v2 genes and to further confirm their expression levels. The RT-qPCR results showed that CYP716A47 was most highly expressed in the flower, while CYP716A53v2 was the most highly expressed in the rhizome and the root. These findings were consistent with the RPKM values (Figure 7A,B). Total of 52 highly expressed UGTs (FPKM ≥ 10) were found in the *P. notoginseng* transcriptome (Supplementary Figure S3). 

### 2.7. Correlation Analysis of Saponin Content and Gene Expression

Pearson correlation analysis was conducted by SPSS 17.0 and the data showed that two CYP gene expression levels had significant correlations with saponin contents (Table 2). The CYP716A47 expression was significantly correlated with the contents of ginsenoside Rc (R = 0.580, *p* < 0.05) and Rb2 (R = 0.707, *p <* 0.01). The CYP716A53v2 expression was associated with the contents of notoginsenoside R1 (R = 0.956, *p <* 0.01), ginsenoside Rg1 (R = 0.991, *p* < 0.01), Re (R = 0.999, *p* < 0.01), Rb1 (R = 0.918, *p <* 0.01), Rd (R = 0.926, *p <* 0.01), PTS (R = 0.994, *p <* 0.01), and total saponins (R = 0.921, *p <* 0.01). These results suggested that CYP716A47 and CYP716A53v2 likely regulated the distribution of protopanaxadiol- and protopanaxatriol-type saponins in the below ground and above ground tissues of *P. notoginseng.*

## 3. Discussion

In this study, the types and contents of saponin components had tissue specificity in the below and above ground tissues of *P. notoginseng*. Protopanaxadiol- and protopanaxatriol-type saponin contents were higher in the below ground tissues than in the above ground tissues, especially in the rhizome. The transcripts were different in the below ground and above ground tissues of *P. notoginseng*. A total of 482 and 882 unigenes were shared in the below ground and above ground tissues, respectively. A total of 38 and 37 CYP transcripts (RPKM ≥ 10) were highly expressed in the below ground and above ground tissues, respectively. The expression level of CYP716A47 was high in the above ground tissues, especially in the flower, and the relative expression of CYP716A53v2 was high in the below ground tissues, especially in the rhizome. The contents of most saponin were remarkably related to the expression levels of CYP716A47 and CYP716A53v2, suggesting that the different expression of CYP716A subfamily genes (CYP716A47 and CYP716A53v2) resulted the specific saponin distribution in the different tissues of *P. notogonseng*.

Ginsenosides have uneven distribution and accumulation in *Panax* plants [8,24]. The levels of ginsenosides in berries are higher than those in *P. ginseng* roots [25]. The total ginsenoside contents in *P. quinquefolius* leaves are higher than those in its roots [26]. In our studies, the contents of saponins were higher in the below ground tissues than in the above ground tissues, especially in the rhizome, and these findings were similar to previous studies [27]. The rhizome is an important part of perennial plants, such as *P. notoginseng*, because it possesses the buds for sprouting in the succeeding year. The abundant accumulation of various saponins in the rhizome can play a defensive role against pathogens and insects. In addition, the contents of protopanaxadiol- (Rb1 and Rd) and protopanaxatriol-type (R1, Rg1, and Re) saponins were higher in the below ground tissues than those in the above ground tissues, whereas protopanaxadiol-type saponins (Rb2 and Rc) were detected in the above ground tissues only. These results were similar to those in previous studies [15,28]. Therefore, saponin types and contents had an uneven distribution in different tissues of *P. notoginseng*.

The transcripts in the below ground and above ground tissues of *P. notoginseng* differed. A total of 1351; 1724; 2042; 4668; 5206; and 12,038 unigenes were specifically expressed in the stem, rhizome, leaf, root, flower, and fibril, respectively, suggesting that the transcripts in the six tissues were differentially expressed. This observation was similar to previous findings [29]. These annotations and transcript differences provided useful information for investigating the functions and expression patterns of unigenes in *P. notoginseng.* A majority of the sequences involved in the carbohydrate metabolic processes were identified in *P. ginseng* [30]. In our data, 55,205 sequences were assigned to the metabolic pathways, and 2128 sequences were annotated to the metabolism of terpenoids and polyketides. Unigene expression has tissue-specific distribution in the roots, stems, leaves, and flowers of *P. ginseng*, and this finding was similar to our results [31]. Consistent with a previous study, our study demonstrated that several transcripts encoding HMGR, MGS, PMK, and MVK were highly expressed in the flowers [31]. The gene-related saponin synthesis showed high expression profiles in the above ground tissues of *P. notoginseng*. Five genes (i.e., FPS, SS, SE1, SE2 and DS) related to saponin biosynthesis were highly expressed in the above ground tissues, especially in the flower [13]. The high contents of saponins were detected in the below ground tissues of *P. notoginseng* in our study. The intermediate products of saponin synthesis may be actively synthesized in above ground tissues and transported to the below ground tissues [32], which revealed that contents of saponins were higher in the below ground tissues of *P. notoginseng*.

The expression patterns of CYP716A47 and CYP716A53v2 genes could regulate the distribution of protopanaxadiol- and protopanaxatriol-type saponins in the different tissues of *P. notoginseng*. CYP716 subfamily genes (CYP716A47 and CYP716A53v2) are involved in the dammarane-type triterpenoid production. CYP716A47 catalyzes the formation of protopanaxadiol from dammarenediol-II [21], and CYP716A53v2 catalyzes the formation of protopanaxatriol from protopanaxadiol in *P. ginseng* [22]. Previous studies revealed that CYP716A47 and CYP716A53v2 are highly expressed in the main root and the rhizome, especially in the periderm [30,33]. The expression level of CYP716A53v2 is higher in *P. notoginseng* roots than in its leaves and flowers [29], and this observation was consistent with our findings. The RNAi of CYP716A47 in the transgenic hairy root of *P. ginseng* and *P. quinquefolius* caused the reduction of ginsenoside synthesis, indicating the positive correlation between gene expression levels and ginsenoside contents [34]. In our study, CYP716A47 expression levels had a significantly positive correlation with the contents of protopanaxadiol-type saponins (Rc and Rb2). CYP716A53v2 gene expression levels had a significantly positive correlation with the contents of protopanaxadiol-type (Rb1 and Rd) and protopanaxatriol-type saponins. These results demonstrated that the expression patterns of CYP716A47 and CYP716A53v2 genes could regulate the distribution of protopanaxadiol-type and protopanaxatriol-type saponins in different tissues of *P. notoginseng*.

Ginsenoside Ro, oleanane-type saponin, was first isolated from *P. japonicus* and also found in other *Panax* species, such as *P. ginseng*, *P. zingiberensis*, and *P. stipuleanatus* [35,36]. Ginsenoside Ro was not detected in our *P. notoginseng* samples. One gene encoding beta-AS (Unigene 20794_All) and one gene encoding CYP716A52v2 (CL11590.Contig2_All) were annotated in our transcript data. Previous studies reported that CYP716A52v2, which served as a CYP716A subfamily gene, may be involved in the oleanane-type triterpene biosynthesis [23]. Similar to a previous study, our study indicated that the content of ginsenoside Ro might be too low to be detected in *P. notoginseng* [29].

## 4. Materials and Methods

### 4.1. Plant Samples

Nine plants of 3-year-old *P. notoginseng* were collected from Wenshan, Yunnan Province in China (23.5° N and 104° E) at their flowering stage in our plantation. *P. notoginseng* seedlings were separated into six different tissues, namely, root, rhizome, fibril, stem, leaf, and flower (Supplementary Figure S4). All voucher specimens were carefully washed, cut into small pieces, and immediately deposited at −80 °C for further processing. Six plants were used for chemical analysis, and other three plants were utilized for molecular experiment.

### 4.2. Saponin Extraction and HPLC analysis

Standards of saponins (i.e., Rg1, Re, Rd, Rb1, Rb2, Rc, and R1) were purchased from Shanghai Tauto Biotech Company (Shanghai, China). These standards have more than 98.0% purity, and their numbers are 16,042,724; 160,907; 160,924; 160,930; 160,606,121; 16,081,931; and 160,923, respectively. The standard stock solutions were dissolved with methanol (Analytical Grade, Fisher, Hampton, NH, USA).Saponins were extracted through ultrasonic extraction in accordance with previously described methods [15]. In brief, the samples were crushed, and 0.1 g of each sample was soaked in 1.0 mL of methanol solution. After more than 10 s of vortexing and 10 min of sonication, the mixture was frozen at −20 °C for 1 h and then centrifuged at 10,000 rpm for 10 min. The upper layer was collected, filtered through a 0.45 µm organic micropore filter, and transferred for HPLC analysis.

The samples were analyzed with an Agilent HPLC 1260 series system (Agilent, Santa Clara, CA, USA) equipped with a VWD detector, a quaternary pump, a column compartment, and an autosampler. A C_18_ reversed phase Eclipse XDB column (5 μm, 4.6 mm × 250 mm, Agilent, Santa Clara, CA, USA) was used for the separation. The gradient was composed of acetonitrile (A) and water (B), and the linear gradient was set as follows in accordance with the Pharmacopoeia of the People’s Republic of China: 0–12 min for 19% A and 12–60 min for 19% A to 36% A. The following parameters were also used: sample injection volume of 10 µL, wavelength of 203 nm, column temperature of 25 °C, and flow rate of 1.0 mL·min^−1^.

### 4.3. RNA Extraction, Library Construction, and RNA Sequencing

Total RNA was isolated from *P. notoginseng* tissues by using a polysaccharide plant polyphenol quick RNA extraction kit (Bio Teke Corporation, Beijing, China) in accordance with the manufacturer’s instructions. Three biological replicates were prepared. The integrity and quantity of RNA were assessed and determined by conducting gel electrophoresis and using a NanoDrop 2000 spectrophotometer (Thermo Fisher Scientific, Hampton, NH, USA), respectively. Transcriptome libraries for RNA sequencing were constructed, detected with Agilent 2100 Bioanalyzer (Agilent, Santa Clara, CA, USA) and ABI Step One Plus Real-time PCR System (Thermo Fisher Scientific, Hampton, NH, USA), and sequenced using an Illumina HiSeq 4000 platform (Illumina, San Diego, CA, USA) to obtain 150 bp paired-end reads.

### 4.4. Transcriptome Analysis

The raw sequencing reads were filtered to obtain the clean reads of each sample. Three criteria were used to filter these reads: adaptors, more than 10% ”N” bases, low-quality reads more than 50% of bases having a qualtiy value ≤ 10. All the sequences obtained from the transcriptome were submitted to NCBI (Accession Number: SRR7427745-SRR7427748). *De novo* assembly from the clean reads of all the samples was conducted using Trinity with default parameters (The completeness of our assembly was evaluate by BUSCO and test result show the complete BUSCO were 88.7%.) (Supplementary Table S6) [37,38]. Unigenes were aligned through BLASTX with the best similar hit of an E-value < 1 × 10^−5^, including NCBI non-redundant (Nr) protein (ftp://ftp.ncbi.nih.gov/blast/db/FASTA), NCBI (ftp://ftp.ncbi.nih.gov/ blast/db), and SWISS-PROT (http://www.uniprot.org/). Homology similarity was searched in the databases, including NCBI (ftp://ftp.ncbi.nih.gov/blast/db) and SWISS-PROT (http://www.uniprot.org/), through BLASTX by using the best similar hit of an E-value < 1× 10^−5^. The classification of protein function was searched in the Gene Ontology (GO) database (http://www.geneontology.org/) and the Clusters of Orthologous Groups (COG) database (www.ncbi.nlm.nih.gov/COG). The Kyoto Encyclopedia of Genes and Genomes (KEGG) pathway database (http://www.genome.jp/kegg/keggl/.html) was used to determine the biological pathways of the unigenes.

The fragments per kilobase of exon model per million mapped reads (FPKM) were calculated to quantify the expression level among the six tissues of *P. notoginseng*. The false discovery rate (FDR) was used to compute the differences in the significance of transcript abundance. log^2^ ratio ≥ 2 and FDR ≤ 0.05 were considered to identify differentially expressed genes (DEGs) (Differential expression analysis were carried out at the gene level.). Heatmaps were constructed using *R* programming language and software (R 3.4.2, R packages).

### 4.5. Real-Time qPCR Analysis of CYP716A47 and CYP716A53v2

The total RNA from *P. notoginseng* tissues was converted into a single-stranded cDNA by using a FastQuant RT kit with gDNase (TIANGEN, Beijing, China). Quantitative reactions were performed using a real-time qPCR system. The reaction mixture contained 10 µL of SYBR Green qPCR Master Mix (Toyobo, Osaka, Japan), 0.7 µL of 10 µM each of the forward and reverse primers, and 2 µL of the cDNA template. Sterile water was added to obtain a final volume of 20 µL. PCR amplification was conducted under the following conditions: 95 °C for 30 s, followed by 40 cycles of 95 °C for 5 s, 55 °C for 15 s, and 72 °C for 15 s, and a dissociation stage of 55 °C for 15 s and 95 °C for 15 s. All primers used in this study were listed in the Supplementary Table S7. The relative expression levels of the key genes were calculated using the 2^−ΔΔ^*C*_t_ method [39].

## 5. Conclusions

In this study, the types and contents of saponin and the transcripts in the below ground and above ground tissues had tissue specificity. Protopanaxadiol- and protopanaxatriol-type saponins were higher in the below ground tissues than those in the above ground tissues. The transcripts were different in the below ground and above ground tissues of *P. notoginseng*. Among 38 and 37 CYP transcripts were highly expressed in the below ground and above ground tissues, respectively. Pearson’s analysis showed that CYP716A47 and CYP716A53v2 could regulate protopanaxadiol- and protopanaxatriol-type saponin distribution in different tissues of *P. notoginseng*.

## Figures and Tables

**Table 1:** Summary of the transcripts and the assembly results for *P. notoginseng.*

Item	No. of Sequences
Clean reads (M)	804.6882
clean bases (G)	120.7032
No. Of contig > 500 bp	1,325,735
Total unigenes	158,551
Total length (bp)	202,104,779
Average contig size (bp)	1274
N50 contig size (bp)	2012
GC (%)	41.05

**Table 2:** Correlation analysis between the expression of two CYP genes and saponins contents.

Genes	NG-R1	G-Rg1	G-Re	G-Rb1	G-Rc	G-Rb2	G-Rd	PTS	PDS	Total Saponins
CYP716A47	−0.056	−0.368	−0.316	−0.153	0.580^*^	0.707^**^	−0.217	−0.338	0.334	−0.103
CYP716A53v2	0.956 **	0.991 **	0.999 **	0.918 **	−0.579 *	−0.596 **	0.926 **	0.994 **	0.454	0.921 **

*. Correlation is significant at the 0.05 level (2-tailed); **. Correlation is significant at the 0.01 level (2-tailed).

## Supplementary Materials

Supplemental data for this manuscript can be found online.

## Abbreviations

AACT	Acetoacetyl-CoA acyltransferase
CYP	Cytochrome
DS	Dammarenediol-II synthase
DXPS	1-deoxy-*O*-xylulose 5-phosphate synthase
DXPR	1-deoxy-*O*-xylulose 5-phosphate reductoisomerase
FPS	Farnesyl pyrophosphate synthase
FPP	Farnesyl diphosphate
FPKM	Fragments per kilobase of exon model per million mapped reads
GDPS	Gerenyl diphosphatesynthase
GO	Gene Ontology
HPLC	High-performance liquid chromatography
HMGS	3-hydroxy-3-methylglutaryl-CoA synthase
HMGR	3-hydroxy-3-methylglutaryl-CoA reductase
HDS	1-hydroxy-2-methyl-2-(E)-butenyl 4-diphosphate synthase
KEGG	Kyoto Encyclopedia of Genes and Genomes
IPPI	Isopentenyl pyrophosphate isomerase
ISPD	2-C-methylerythritol 4-phosphatecytidyl transferase
ISPE	4-(cytidine-5′-diphospho)-2-C-methylerythritol kinase
IPP	Isoprenyl diphosphate
ISPH	1-hydroxy-2-methyl-2-(E)-butenyl 4-diphosphate reductase
MVDD	Mevalonate diphosphate decarboxylase
MECPS	2-C-methylerythritol-2,4-cyclophosphate synthase
MEP	2-C-methyl-d-erythritol-4-phosphate
MVA	Mevalonate
MVK	Mevalonate kinase
NCBI Nr	NCBI Non-redundant protein
ORF	Open read frame
PTS	20(S)-protopanaxatriol saponins
PDS	20(S)-protopanaxadiol saponins
PMK	Phosphomevalonate kinase
SS	Squalene synthase
SE	Squalene epoxidase
UGTs	UDP-glycosyltransferases
