# Supplementary material for: Integrated Chemical and Transcriptomic Analysis Reveals the Distribution of Protopanaxadiol- and Protopanaxatriol-Type Saponins in Panax notoginseng
Source: Molecules. 2018 Jul 19;23(7):1773. doi: 10.3390/molecules23071773 (PMC6099965; doi:10.3390/molecules23071773)

***Supplementary data***

Integrated Chemical and Transcriptomic Analysis Reveals the Distribution of Protopanaxadiol- and Protopanaxatriol-Type Saponins in *Panax notoginseng*

Guangfei Wei ^1,2^, Fugang Wei ^3^, Can Yuan ^2^, Zhongjian Chen ^4^, Yong Wang ^4^, Jiang Xu ^2^, Yongqing Zhang ^1^, Linlin Dong ^2^*, Shilin Chen ^2,^*

^1^ Shandong University of Traditional Chinese Medicine, Jinan 250355, China,

^2^ Key Laboratory of Beijing for Identification and Safety Evaluation of Chinese Medicine, Institute of Chinese Materia Medica, China Academy of Chinese Medical Sciences, Beijing 100700, China

^3^ Wenshan Miaoxiang Notoginseng Technology Co., Ltd., Wenshan 663000, China.

^4^ Institute of Sanqi Research, Wenshan University, Wenshan 663000, China

Correspondence: lldong@icmm.ac.cn (L.L.D.); slchen@icmm.ac.cn (S.L.C.); Tel: 0086-10-57203877; Fax: 0086-10-62899776;

**Supplementary Table 1** Calibration curves, linearity, precision, repeatability, stability and recovery rate of seven saponins of notoginsenoside R1 and six major saponins (i.e., Rg1, Re, Rc, Rd, Rb1, Rb2).

| Saponins | Calibration curves | *R^2^* | Linearity（ug） | RSD% (*n*=5) | | | Recovery rate (%) (RSD%) |
| --- | --- | --- | --- | --- | --- | --- | --- |
|  |  |  |  | Precision | Repeatability | Stability |  |
| NG-R1 | *Y*=236.44*X*+36.899 | 0.9997 | 0.18-3.6 | 0.1 | 2.82 | 1.87 | 99.25(2.02) |
| G-Rg1 | *Y*=237.42*X*+15.767 | 0.9993 | 0.26-5.2 | 0.12 | 1.08 | 2.71 | 98.44(2.60) |
| G-Re | *Y*=398.59*X*+6.1494 | 0.9992 | 0.07-1.4 | 0.08 | 2.01 | 2.09 | 102.44(1.26) |
| G-Rb1 | *Y*=227.91*X*+2.0157 | 0.9998 | 0.29-5.8 | 0.35 | 0.17 | 2.82 | 100.47(0.51) |
| G-Rc | *Y*=181.14*X*+3.0806 | 0.9993 | 0.099-1.98 | 0.58 | 0.93 | 2.42 | 100.69(1.18) |
| G-Rb2 | *Y*=231.26*X*+3.6762 | 0.9999 | 0.094-1.88 | 1.00 | 2.58 | 2.64 | 96.78(2.51) |
| G-Rd | *Y*=235.94*X*+7.4172 | 0.9999 | 0.16-3.2 | 0.08 | 0.83 | 1.18 | 99.81(1.64) |

The linearity of all calibration curves were *R*^2^ > 0.999. The precision variations, repeatability variations and stability variations were 0.08%–1.00%, 0.83%–2.82%, and 1.18%–2.82%, respectively. The recoveries ranged from 96.78% – 102.44%, with variations of 0.51%–2.60%. These results showed that the established HPLC method was accurate and sensitive, which was appropriate for the quantitative analyses.

**Supplementary Table 2**. Statistics of transcriptome data for *P. notoginseng* different parts.

| **Tissues** | **Duplicates** | **No. of**  **clean reads (M)** | **No. of**  **clean bases (G)** | **Read length (bp)** | **Q20 (%)** | **Q30 (%)** | **GC (%)** |
| --- | --- | --- | --- | --- | --- | --- | --- |
| Root | Root-1 | 45.3693 | 6.8054 | 150 | 98.39 | 95.84 | 43.18 |
|  | Root-2 | 44.9932 | 6.7490 | 150 | 96.26 | 91.33 | 45.09 |
|  | Root-3 | 44.4953 | 6.6743 | 150 | 96.28 | 91.40 | 44.55 |
| Fibril | Fibril-1 | 43.3008 | 6.4951 | 150 | 95.84 | 89.94 | 44.44 |
|  | Fibril-2 | 44.9282 | 6.7392 | 150 | 96.30 | 91.44 | 44.13 |
|  | Fibril-3 | 44.2562 | 6.6384 | 150 | 96.24 | 91.30 | 44.51 |
| Rhizome | Rhizome-1 | 44.9970 | 6.7496 | 150 | 96.13 | 91.10 | 45.20 |
|  | Rhizome-2 | 44.7416 | 6.7112 | 150 | 95.92 | 90.66 | 45.06 |
|  | Rhizome-3 | 44.7973 | 6.7196 | 150 | 96.09 | 91.00 | 44.93 |
| Stem | Stem-1 | 44.9111 | 6.7367 | 150 | 96.16 | 91.12 | 45.67 |
|  | Stem-2 | 44.8734 | 6.7310 | 150 | 96.21 | 91.21 | 45.65 |
|  | Stem-3 | 44.7262 | 6.7089 | 150 | 96.08 | 90.94 | 45.50 |
| Leaf | Leaf-1 | 44.1505 | 6.6226 | 150 | 96.13 | 91.07 | 44.79 |
|  | Leaf-2 | 45.1217 | 6.7683 | 150 | 96.21 | 91.23 | 44.70 |
|  | Leaf-3 | 44.0934 | 6.6140 | 150 | 96.13 | 91.08 | 44.27 |
| Flower | Flower-1 | 44.8985 | 6.7348 | 150 | 96.49 | 91.84 | 44.41 |
|  | Flower-2 | 44.9577 | 6.7436 | 150 | 96.31 | 91.45 | 44.86 |
|  | Flower-3 | 45.0768 | 6.7615 | 150 | 96.23 | 91.30 | 44.90 |
| Total | 18 | 804.6882 | 120.7032 | 150 | - | - | - |

**Supplementary Table 3.** The up-regulated and down-regulated DEGs in different parts of *P. notoginseng* (FDR ≤ 0.05 & FC ≥ 2 ).

| **Item** | **Up-Regulated** | **Down-Regulated** | **Total** |
| --- | --- | --- | --- |
| Fibril-VS-Rhizome.DEseq2 | 6796 | 7187 | 13983 |
| Fibril-VS-Root.DEseq2 | 5633 | 5041 | 10674 |
| Flower-VS-Fibril.DEseq2 | 9965 | 12964 | 22929 |
| Flower-VS-Leaf.DEseq2 | 5918 | 7730 | 13648 |
| Flower-VS-Rhizome.DEseq2 | 9145 | 12752 | 21897 |
| Flower-VS-Root.DEseq2 | 6658 | 7427 | 14085 |
| Flower-VS-Stem.DEseq2 | 6898 | 12476 | 19374 |
| Leaf-VS-Fibril.DEseq2 | 8370 | 8806 | 17176 |
| Leaf-VS-Rhizome.DEseq2 | 8109 | 8263 | 16372 |
| Leaf-VS-Root.DEseq2 | 6310 | 6514 | 12824 |
| Rhizome-VS-Root.DEseq2 | 423 | 815 | 1238 |
| Stem-VS-Fibril.DEseq2 | 9978 | 9701 | 19679 |
| Stem-VS-Leaf.DEseq2 | 7486 | 7633 | 15119 |
| Stem-VS-Rhizome.DEseq2 | 6409 | 6831 | 13240 |
| Stem-VS-Root.DEseq2 | 6519 | 6263 | 12782 |

**Supplementary Material 4: Supplementary Table 4.** The average expression level of unigenes in *P. notoginseng.*

**Supplementary Material 5: Supplementary Table 5.** Candidate 175 transcripts involved in ginsenosides biosynthesis and their expression level in *P. notoginseng* (RPKM>10).

**Supplementary Material 6: Supplementary Table 6.** BUSCO values.

**Supplementary Table 7.** List of real-time PCR primer sequences

| **Gene names** | **Primer(5'-3')** | |
| --- | --- | --- |
|  | **Forward** | **Reverse** |
| CYP716A47 | GGATGTGCCCTGGGAGTG | GCGTACAAGGTGATAGACGAATAG |
| CYP716A53v2 | ACAATCACCTTCGTTATCAACTATC | TCTTCCTCAAATCCTCCCAAT |
| 18S rRNA | GATGCGCTCCTGTCCTTAAC | CATCCTTGGCAAATGCTTTC |

**Supplementary Figure 1** Venn diagram of the homology and protein sequence similarity from transcriptome analysis.


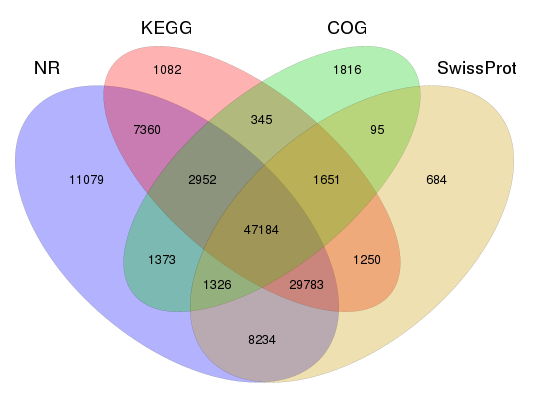


**Supplementary Figure 2.** Functional classsification of unigenes. Based on Gene Ontology categories(A) and Orthologous Groups (B), respectively.


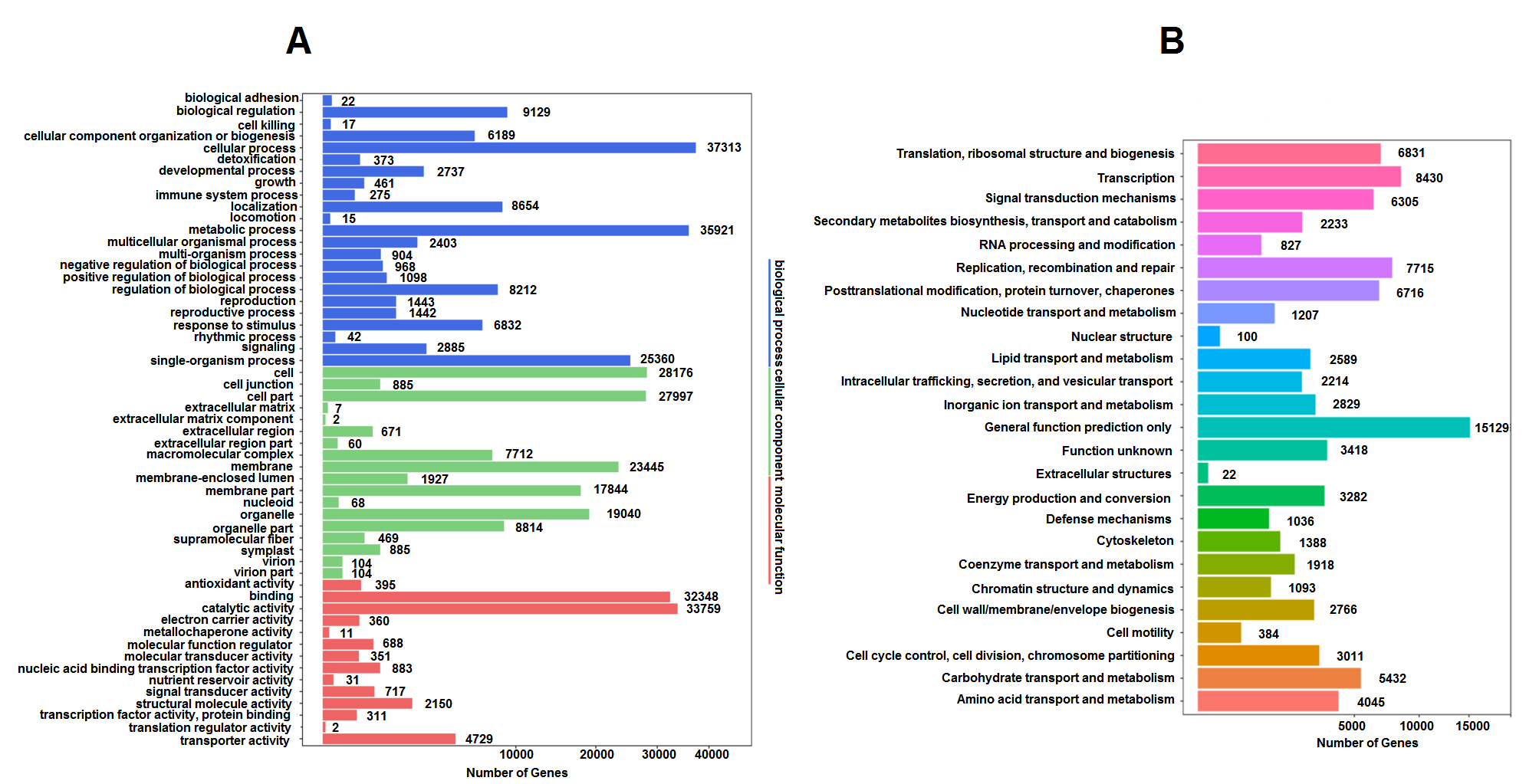


**Supplementary Figure 3.** Heatmap of UGTs genes involved in saponins biosynthesis in *P. notoginseng* using an average value of three duplicates. (FPKM≥10). UGTs are known to catalyze glycosylation in the last step of saponins biosynthesis. Total of 52 highly expressed UGTs (RPKM ≥ 10) were found in the *P. notoginseng* transcriptome. Among the most highly-abundant transcripts, the abundance of 13 UGTs were more highly expressed in the underground parts (rhizome and root) than that in aerial parts. 10 UGTs were expressed highest in the flower. 11 UGTs were highly expressed in the leaf. Multiple highly-expressed CYPs and UGTs suggested complex regulatory mechanisms of various saponin distribution in *P. notogisneg*.


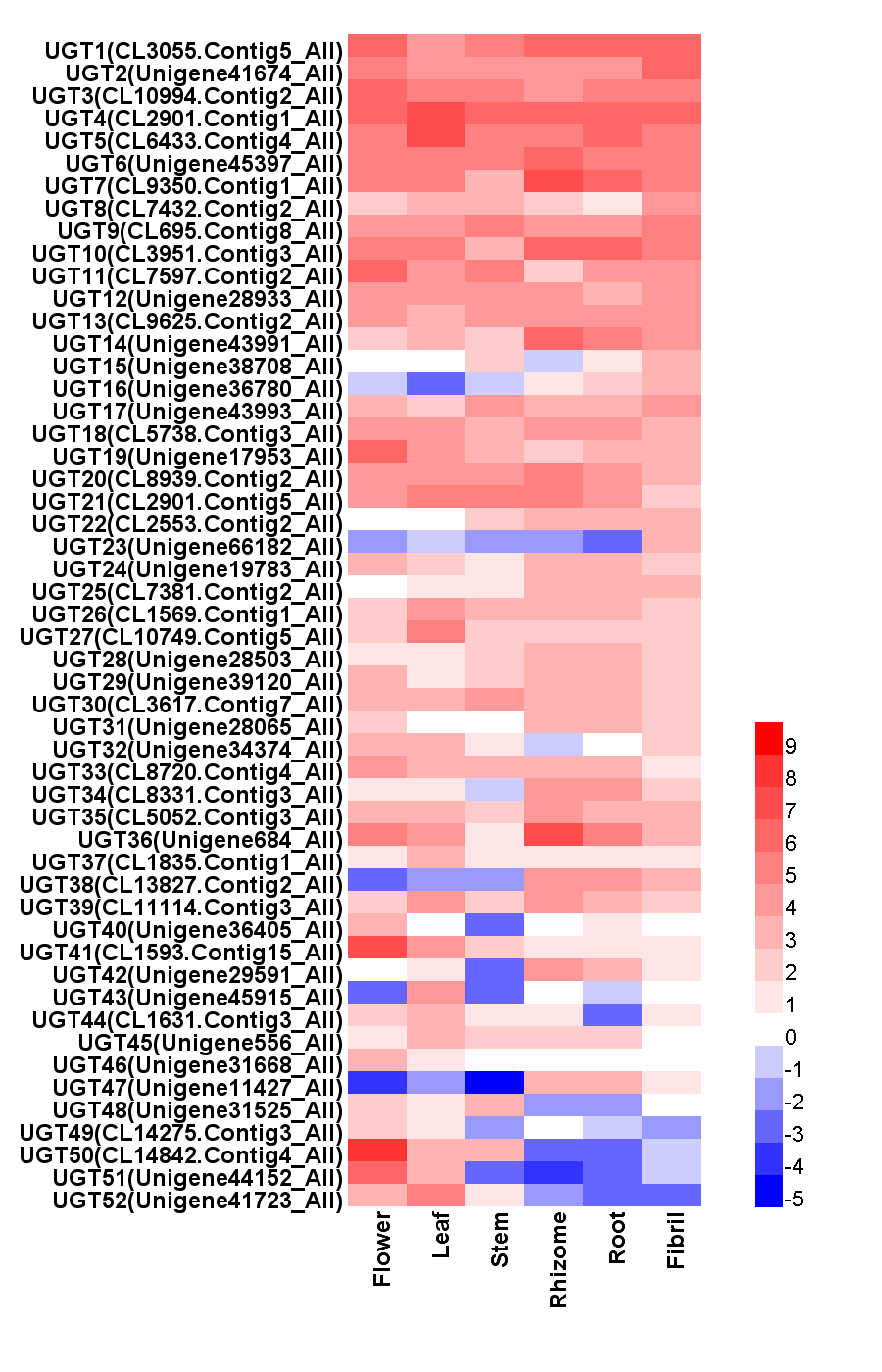


**Supplementary Figure 4**. Plant parts of *P. notoginseng* seedling for experiments. (A) Stem; (B) Leaf; (C) Flower; (D) Root; (E) Rhizome; (F) Fibril. Above ground tissues include (A) Stem; (B) Leaf; (C) Flower. Below ground tissues include (D) Root; (E) Rhizome; (F) Fibril.


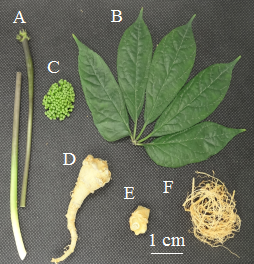

Supplement: Supplementary file 1 [file molecules-23-01773-s001.zip › New folder/molecules-331747-supplementary material .docx]
